# Regeneration of temporomandibular joint using in vitro human stem cells: A review

**DOI:** 10.1002/term.3302

**Published:** 2022-03-31

**Authors:** Shan Gong, Chitra Priya Emperumal, Kamal Al‐Eryani, Reyes Enciso

**Affiliations:** ^1^ Master of Science Program in Orofacial Pain and Oral Medicine Herman Ostrow School of Dentistry of University of Southern California Los Angeles California USA; ^2^ Division of Periodontology, Diagnostic Sciences & Dental Hygiene Herman Ostrow School of Dentistry of University of Southern California Los Angeles California USA; ^3^ Department of Geriatrics, Special Needs and Behavioral Sciences Herman Ostrow School of Dentistry of University of Southern California Los Angeles California USA

**Keywords:** in vitro, mesenchymal stem cells, review, stem cell therapies, temporomandibular disorders, temporomandibular joint

## Abstract

Temporomandibular joint disorders (TMDs) range from gross anatomic deformities of the disc and hard tissue to functional disturbances. Traditional treatment of TMDs includes physical therapy, use of appliances, pharmacological, surgical and psychological interventions. However, during the late stage of TMDs, conventional management often results in inadequate relief of symptoms. Stem cell‐based tissue regeneration has been studied extensively in joint regeneration, including the Temporomandibular Joint (TMJ). This study aims to review the potential of various human stem cells (HSC) for the regeneration of the TMJ. In vitro studies using human mesenchymal stem cells cultured under different conditions to evaluate regeneration of TMJ related structures were searched on PubMed, EMBASE, Cochrane, and Web of Science up to March 2020. In vitro studies utilized several different types of stem cells under varying conditions. Increased osteogenesis and/or chondrogenesis were noted with stem cell interventions compared to control groups on Alkaline Phosphatase (ALP) activity, Col‐I, Col‐II, Col‐X, RUNX2, LPL, and Aggrecan mRNA expression. This review emphasizes the potential of stem cell therapies in the regeneration of TMJ‐related structures. However, further in vivo studies are required to evaluate the efficacy and safety of these therapies in humans.

## 1 INTRODUCTION

**FIGURE 1:**
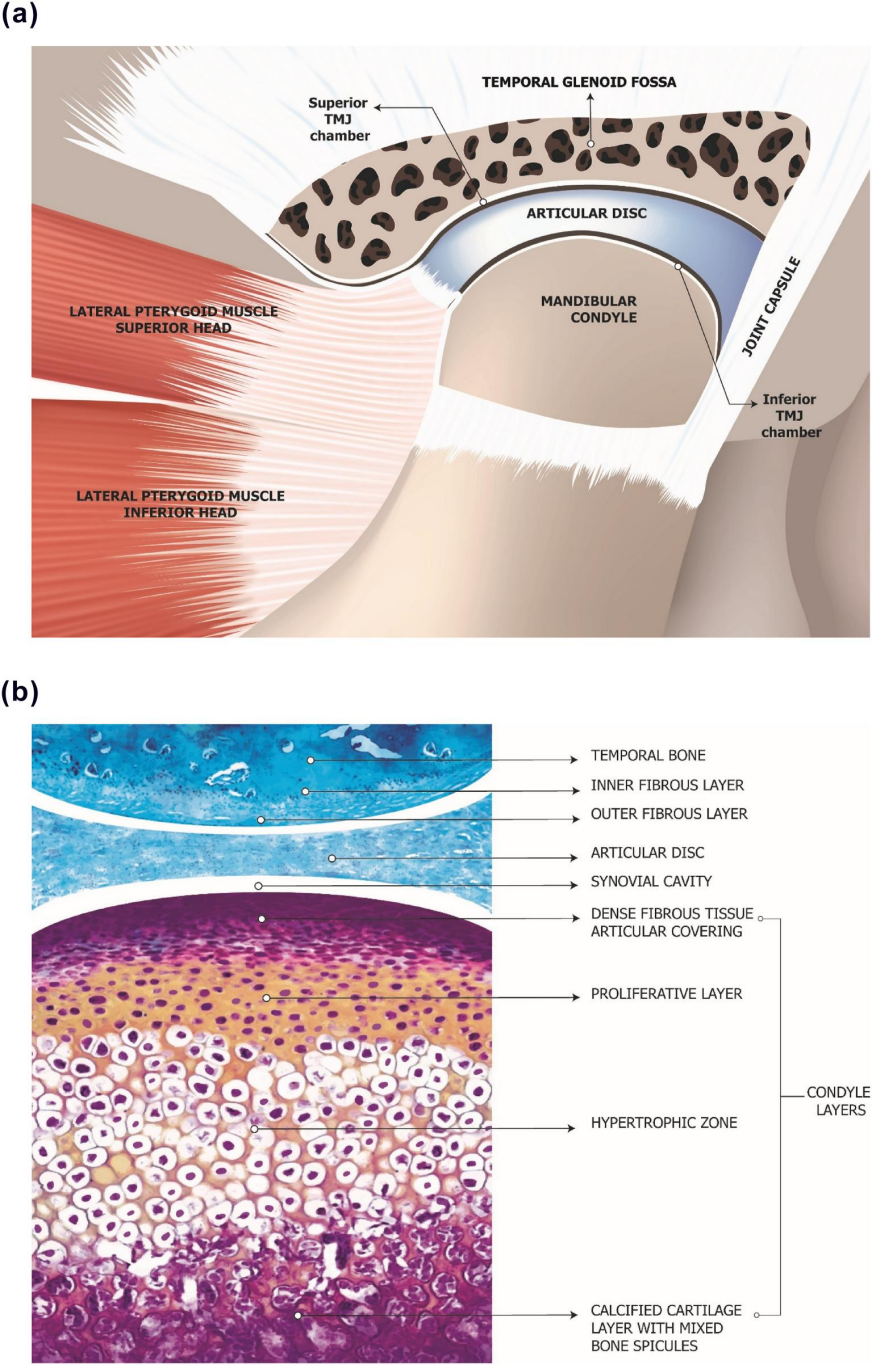
(a) Anatomy of the TMJ. (b) Histological structure of TMJ including temporal bone, the articular disk and head of condyle

The temporomandibular joint (TMJ) is a complex synovial joint with a unique articulation between the temporal glenoid fossa and the mandibular condyle, which are separated by an articular disc composed of avascular and non‐innervated dense fibrous connective tissue with a varying amount of fibrocartilage. The articular disc divides the TMJ cavity into upper and lower chambers. Gliding movement occurs in the upper chamber during the maximal mouth opening, while the lower chamber function primarily as a hinge or rotary movement in the early opening. Because the TMJ has a hinge and sliding movable socket, it is classified as ginglymoarthrodial a joint. The TMJ is surrounded by a fibrous connective tissue capsule attached to muscles and tendons. The capsule is lined by a synovium membrane that secretes the lubricating synovial fluid (Piette, 1993) (Figure 1a).

The development of the TMJ is different from other joints in that the cartilage of the mandible's condyle is secondary cartilage compared to the articular cartilage found in other joints, which is primary cartilage associated with endochondral ossification that is dominated by hyaline cartilage. Secondary cartilage develops in association with specific bones formed by intra‐membranous ossification after the bones are already formed. These developing bones become entirely surrounded by the periosteum, including the areas that eventually form the articular surfaces of the TMJ. The periosteum lining these articular surfaces is gradually transformed into the dense fibrous articular tissues of the TMJ during its early development (Symons, 1965). Mature condylar cartilage consists of four zones: (1) **superficial** or articular zone of fibrous tissue facing the disc expressing collagen I, (2) **proliferative** pre‐chondroblastic zone expressing collagen I, (3) a **chondroblastic** zone expressing collagen II, the proteoglycans aggrecan, decorin, chondroitin sulphate PG, and keratin sulphate PG, and (4) a **hypertrophic** zone adjacent to bone expressing collagen X. Therefore, TMJ has a unique hybrid structure integrating a superficial fibrocartilage layer covering a secondary hyaline cartilage layer (Chen et al., 2020). It is critical for regenerative cell‐based therapies specific for the TMJ to reproduce the zonal architecture of the mandibular condylar cartilage (Chen et al., 2020); however, one of the major challenges for developing an effective regeneration therapy is the lack of understanding of the unique formation of the condylar cartilage from the other synovial joints and replicate this zonal architecture (Tanaka & Koolstra, 2008) (Figure 1b).

Temporomandibular disorders (TMDs) include arthralgia, localized myalgia, myofascial pain, internal derangements (disc displacement with or without reduction), degenerative joint diseases, subluxation, and headache attributed to TMD (Schiffman et al., 2014). Conservative measures, such as, physical therapy, oral appliance therapy, pharmacotherapy including topical and systemic medications, glucocorticosteroid injections and arthrocentesis could help in the early stages of repair (Dantas & Vivan, 2015; Dimitroulis et al., 1995; Durham et al., 2015). As the degeneration progresses, bony erosion, condylar resorption, and discal rupture/deformation can be seen radiographically (Brooks et al., 1997). When the wear goes beyond repair, in conditions such as severe trauma or systemic conditions (including autoimmune arthritis, connective tissue disorders and idiopathic condylar resorption), patients may need invasive treatment, involving open surgeries with replacement of the whole or parts of the TMJ with autogenous or allogenous materials (Elgazzar et al., 2010). The prognosis of surgeries varies, depending on the severity of the condition, co‐morbidities related to patient's health and the practitioners' knowledge and skills (Dimitroulis, 2005).

Damage to TMJ structures is usually irreversible, and commonly used treatment strategies described above cannot restore damaged TMJ tissues. Stem cell‐based therapy is sought as an alternative approach to current treatment strategies, according to few recent studies, to repair discal or bony damage from common conditions such as rheumatoid arthritis and osteoarthritis (Cui et al., 2017; Serakinci & Savtekin, 2017). Two main types of stem cells exist: omnipotent embryonic and non‐embryonic/adult stem cells (Helgeland et al., 2018), which include hematopoietic and mesenchymal stem cells (MSCs), along with neural, epithelial and skin stem cells. MSCs are multipotent stem cells that can be isolated from many human tissues such as bone marrow, synovium, fat, muscle, and periodontal ligament of teeth (Mao et al., 2006). Cells used for TMJ regeneration include whole bone marrow extracts, synovial‐derived MSCs, bone marrow‐derived, adipose tissue‐derived and fibrocartilage stem cells, chondrocytes, osteoblasts, and fibroblast‐like synoviocytes. Bone marrow‐derived stem cells are most frequently used, either alone or embedded in natural or synthetic polymer scaffolds (Helgeland et al., 2018; Mao et al., 2006; Puelacher, 2011). In addition, various growth factors such as bone morphogenetic protein 2 (BMP‐2), transforming growth factor‐beta 2 (TGF‐beta 2), connective tissue growth factor (CTGF), transforming growth factor‐beta 1 (TGF‐beta 1), and platelet‐rich plasma (PRP) have been used alone or in combination with cells and/or scaffolds to regenerate discal or osteochondral TMJ tissues (Helgeland et al., 2018; Puelacher, 2011). PRP is used most often due to its various growth factors, such as platelet‐derived epidermal growth factor (PD‐EGF), platelet‐derived growth factor (PDGF), bone morphogenetic protein (BMP), transforming growth factor (TGF), insulin‐like growth factor (IGF), vascular endothelial growth factor (VEGF), endothelial cell growth factor (ECGF), basic fibroblast growth factor (bFGF) (Coskun et al., 2019; Foster et al., 2009; Kılıç et al., 2019; Wang et al., 2018).

Studies have shown that human MSCs can promote TMJ tissue damage repair, suppress the inflammatory response, and modulate the immune system (El Qashty et al., 2018). Transplanted MSCs can seed in the target tissue or migrate to the target tissue and differentiate into mature cells to help in tissue repair (Cui et al., 2017; Serakinci & Savtekin, 2017). These cells also possess anti‐inflammatory and immunomodulatory properties, which can speed up the healing process in the TMJ (van Poll et al., 2008). Growth factors such as IGF, bFGF, VEGF secreted by stem cells can participate in various levels of tissue regeneration, most importantly, stimulate bone regeneration (Tomoyasu et al., 2007). A few in vivo human studies (Carboni et al., 2019; De Riu et al., 2019; Howlader et al., 2017) have demonstrated the regenerative potential of stem cells in TMJ repair. Carboni et al. (2019) found significant improvement of TMD related symptoms in four patients with internal derangement of TMJ, who received stem cell injections as compared to controls with saline injection only. They also found MRI evidence of restoration of defective TMJ structures in stem cell group. A larger Randomized Clinical Trial (RCT) by De Riu et al. (2019) found that at 6 months and 1‐year follow‐up, patients receiving bone marrow nucleated cells showed significantly better relief of TMD‐related symptoms as compared to controls who received TMJ arthrocentesis only. However, De Riu et al. (2019) did not find any cartilage or bony lesion healing by MRI. Howlader et al. (2017) found significant improvement in patients with TMJ ankylosis with stem cell injection. The results of these studies demonstrate the potential for treatment of TMJ internal derangement with stem cell injections, however due to the variations in the diagnosis and the types of stem cells used, their results were partially conflicting. Better designed clinical trials are needed to confirm the observations of these in vivo studies.

Reviews about TMJ regeneration in experimental animal models have been published recently by Helgeland et al. (2018) and Liu et al. (2014). However, because exogenous MSC injection showed low grafting efficiency, high risk of infection, and the possibility of neoplastic transformation, human clinical trials with stem cells are limited (Cui et al., 2017; Serakinci & Savtekin, 2017). We reviewed current literature regarding human stem cell based TMJ regeneration to assess the efficacy of stem cells for TMJ regeneration/repair in vitro and to explain the necessity for further research for better patient care. Studies involving experimental animal models and/or animal stem cells were excluded due to two recent reviews published on this topic (Helgeland et al., 2018; Liu et al., 2014).

## 2 MATERIALS AND METHODS

### 2.1 Research question

The PICOS question for this research was:

- Study design: In vitro studies
- *Population:* Human stem cells
- *Intervention:* Regeneration therapy with stem cells
- *Comparison:* Other cells
- *Outcomes:* Increased differentiation of stem cells to TMJ‐related structures
- *Setting:* Laboratory setting

### 2.2 Inclusion and exclusion criteria

*Inclusions:* Human stem cells cultured in vitro under different conditions to evaluate regeneration of TMJ‐related structures were included.

*Exclusions:* Any in vivo study, study using animal models, animal stem cells, or not related to TMJ regeneration was excluded.

### 2.3 Search methods for identification of studies

For the identification of studies included or considered for this review, detailed search strategies were developed for each database searched. The search strategy used a combination of MESH terms and free‐text terms. Four electronic databases were searched (MEDLINE via PubMed, Web of Science, Cochrane Library, and EMBASE) up to March 3, 2020 by the senior author (RE) using the strategies reported in Table 1.

**TABLE 1:** Electronic database search strategies

Electronic database	Search strategy
**MEDLINE via PubMed** (searched up to March 3, 2020)	(“Temporomandibular Joint Disorders”[Mesh] OR “Temporomandibular Joint”[MeSH] OR “Temporomandibular Joint Dysfunction Syndrome” [Mesh] OR “Temporomandibular Joint Disc” [MeSH] OR “Temporo‐mandibular” OR “Temporomandibular” OR “TMJ pain” OR “TMJ arthritis” OR “TMJ” OR (temporomandibular joint) OR (temporo‐mandibular joint) OR (temporomandibular disorder*) OR (temporo‐mandibular disorder))** AND** (“Stem Cell Transplantation”[Mesh] OR “Adult Stem Cells”[Mesh] OR “Mesenchymal Stem Cells”[Mesh] OR (stem cell*) OR (bone marrow) OR “Hematopoietic Stem Cells”[Mesh] OR (Hematopoietic stem cell*) OR (Mesenchymal stem cell*) OR “Chondrocytes”[Mesh] OR Chondrocyte* OR “Bone Marrow Transplantation”[Mesh])
	**Filters:** Humans, Clinical Trial, Review, Systematic Reviews, Randomized Controlled Trial
	**Language Filters:** English, Chinese
**The Web of Science** (searched up to March 3, 2020)	TOPIC: ((temporomandibular arthritis) OR (temporomandibular disorder) OR (temporo‐mandibular disorder) OR (Temporomandibular Joint Dysfunction Syndrome) OR (temporomandibular joint) OR (TMJ arthritis))** AND** TOPIC: ((Stem Cell Transplantation) OR (Mesenchymal Stem Cells) OR (Bone Marrow Transplantation) OR (hematopoietic stem cell))
**The Cochrane Library** (searched up to March 3, 2020)	#1: ((temporomandibular arthritis) OR (temporomandibular disorder) OR (temporo‐mandibular disorder) OR (Temporomandibular Joint Dysfunction Syndrome) OR (temporomandibular joint) OR (TMJ arthritis))
	#2: ((Stem Cell Transplantation) OR (Mesenchymal Stem Cells) OR (Bone Marrow Transplantation) OR (hematopoietic stem cell))
	#3: #1 and #2
**EMBASE** (searched up to March 3, 2020)	#1: “temporomandibular joint”/exp OR “temporomandibular joint” OR “temporomandibular joint disorder”/exp OR “temporomandibular joint disorder”
	#2: “stem cell” OR “stem cell transplantation” OR “mesenchymal stem cell” OR “bone marrow cell” OR “hematopoietic stem cell”
	#3: #1 and #2
	#4: #3 AND “human”/de AND (“article”/it OR “article in press”/it OR “conference paper”/it OR “review”/it)
	#5: #4 AND (“case report”/de OR “clinical article”/de OR “clinical trial topic”/de OR: “comparative study”/de OR “evidence based practice”/de OR “human”/de OR “in vivo study”/de OR “major clinical study”/de OR “practice guideline”/de OR “systematic review”/de)

### 2.4 Data extraction and management

One author (S.G.) scanned the articles retrieved from the application of the search strategy, and acquired the full manuscript if the study met the inclusion criteria or when a definite decision could not be made regarding inclusion or exclusion based on the title and abstract only. The reason for exclusion of the studies was also recorded. The full‐text articles were analyzed for inclusion/exclusion, and relevant data was extracted by the same review author (S.G.) using a previously prepared data extraction form. The form from the reviewer was then reviewed by the senior author (R.E.). The form included for each study: the study design, characteristics of the sample (sample size, inclusion/exclusion criteria), interventions, control groups, and outcomes.

### 2.5 Statistical analyses

Results of in vitro studies are presented in tabular form. Estimated **risk ratios** were calculated as the relative expression in the stem cells group divided by the relative expression in the controls for those studies reporting ALP activity, GAG content, Col‐I, relative Col‐I, Col‐II, Col‐X, SOX9, RUNX2, LPL, and Aggrecan mRNA expression in the stem cell‐based interventions and the control groups.

## 3 RESULTS

### 3.1 Results of the search

**FIGURE 2:**
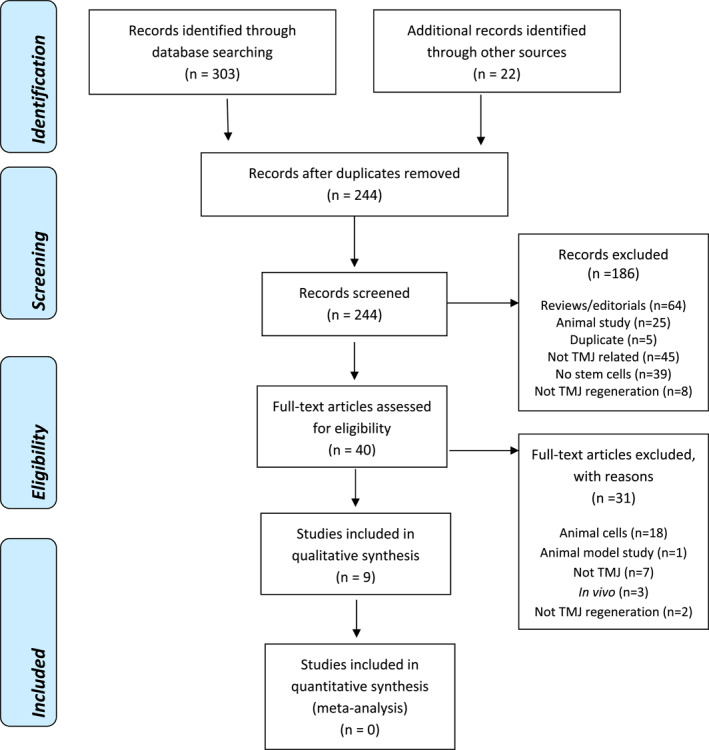
PRISMA flow diagram

Through the preliminary search strategy by database on March 3, 2020, 303 references were found, and further 22 more records were discovered through other sources like searching references of included studies and reviews. After duplicate elimination, 244 references were analyzed individually by review authors (S.G. and R.E.), and based on the abstracts and titles, 186 articles were excluded and 40 articles were included. Of those 186 studies, reasons for exclusion were as follows: reviews/editorials (*n* = 64), animal studies (*n* = 25), duplicates (*n* = 5), not TMJ related (*n* = 45), no stem cells intervention (*n* = 39), not TMJ regeneration (*n* = 8). The full texts of these 40 manuscripts were analyzed for inclusion individually by the same authors, and nine manuscripts were found relevant for inclusion (in vitro). Reasons for exclusions were as follows: the authors used animal cells (*n* = 18), or it was an animal model study (*n* = 1), or not TMJ related (*n* = 7), or not TMJ regeneration (*n* = 2), or in vivo *study* (Carboni et al., 2019; De Riu et al., 2019; Howlader et al., 2017) (*n* = 3). PRISMA flowchart shows a summary of our results (Figure 2).

### 3.2 Study characteristics

In total, nine in vitro studies were identified using HSCs to regenerate TMJ structures with a sample size varying from 3 to 19. The features of included in vitro studies are summarized in Table 2.

**TABLE 2:** Study characteristics of in vitro studies included in this review

	Objective	Study group		Control group		Both
Study	(Ligament, disc, bone)	Type of cells	Type of stimuli	Type of cells	Type of stimuli	Identified mediators
Bailey et al. (2007)	TMJ condylar cartilage regeneration	Human umbilical cord derived stem cells (3.4 × 10^6^/flask)	EGF, PDGF, ITF+1 (chondrogenic medium)	TMJ condylar chondrocytes (cells were plated and fed until confluent)	TGF‐beta1, ITS+1 (control medium)	Polyglycolic acid scaffold
Bousnaki et al. (2018)	TMJ disc regeneration	Dental pulp stem cells (3 × 10^5^/well of 6‐well plate)	None	Human nucleus pulposus cells (10^5^/well of 6‐well plate)	None	Chitosan/alginate scaffold with or without glutaraldehyde
Brady et al. (2011)	Biphasic osteochondral regeneration	Human MSC (2.2 × 10^4^/cm^2^)	MSCs preconditioned in osteogenic or chondrogenic media	No control group		
Koyama et al. (2011)	Multi‐lineage differentiation evaluation	Synovial fluid‐derived adherent cells by arthrocentesis (1 × 10^7^/ml)	Chondrogenic inductive medium: rhBMP‐2; osteogenic induction medium: adipogenic induction medium, neuronal induction medium	Synovial fluid‐derived adherent cells by arthrocentesis (1 × 10^7^/ml)	Control medium	None
Legemate et al. (2016)	TMJ disc regeneration	Human MSC (1 × 10^6^/ml)	50 and 100 mg CTGF/TGF beta 3 containing microspheres	Human MSCs (1 × 10^6^/ml)	No growth factor microsphere.	3D‐printed scaffolds
Liu et al. (2017)	Multi‐lineage differentiation evaluation	Synovial fluid‐derived MSC (500 cells/cm^2^)	IL‐8, IL‐1beta, IL‐6, IL‐10, TNF‐a, IL‐12p cytokines stimulation medium/Osteogenic/Adipogenic/Chondrogenic/Neurogenic induction medium	Synovial fluid‐derived MSC (500 cells/cm^2^)	Culture medium without induction or cytokine stimulation	
Sun et al. (2014)	Multi‐lineage differentiation evaluation	Synovium fragment‐derived MSC; Synovial fluid MSC (5000–10,000/well of 96‐well plates)	Osteogenic/Adipogenic/Chondrogenic/Neurogenic induction medium.	Synovium fragment‐derived MSC; Synovial fluid MSC (5000–10,000/well of 96 well plates)	Control medium	None
Yao et al. (2018)	Multi‐lineage differentiation evaluation	Synovium fragment‐derived MSC from patients with TMJ osteoarthrosis (5000/cm^2^ for osteogenic and adipogenic differentiation; 3 × 10^5^ for chondrogenic differentiation)	Osteogenic/Adipogenic/Chondrogenic differentiation medium	Surgery‐obtained synovium specimens cells (5000/cm^2^ for osteogenic and adipogenic differentiation; 3 × 10^5^ for chondrogenic differentiation)	Osteogenic/Adipogenic/Chondrogenic differentiation medium	None
Zhang et al. (2014)	Fibrocartilage regeneration	Periodontal ligament derived MSC plus TMJ fibrochondrocytes (1 × 10^5^ PD‐MSC and 1 × 10^5^ chondrocytes mixed/well)	None	TMJ fibrochondrocytes alone (2 × 10^5^/well)	None	None

Abbreviations: CTGF/TGF, connective tissue growth factor/transforming growth factor; EGF, epithelial growth factor; IL‐10, interleukin 10; IL‐12p, interleukin 12p; IL‐1b, interleukin 1b; IL‐6, interleukin 6; IL‐8, interleukin 8; ITF+1, insulin transferrin‐selenium plus 1; MSC, mesenchymal stem cell; PDGF, platelet‐derived growth factor; rhBMP‐2, recombinant human bone morphogenetic protein‐2; TGF‐beta 1, human transforming growth factor beta‐1; TMJ, temporomandibular joint; TNF‐a, tumor necrotizing factor alpha.

Brady et al. (2011) and Legemate et al. (2016) used bone marrow derived MSCs in their research. Other stem cells that were studied include human umbilical cord matrix stem cells (Bailey et al., 2007), synovial fluid‐derived stem cells (Koyama et al., 2011; Liu et al., 2017; Sun et al., 2014; Yao et al., 2018), dental pulp stem cells (Bousnaki et al., 2018), and periodontal ligament‐derived MSCs (Zhang et al., 2014).

Legemate et al. (2016) and Bousnaki et al. (2018) cultured stem cells in scaffolds to regenerate TMJ disc. Legemate et al. (2016), Bailey et al. (2007), and Liu et al. (2017) cultured stem cells with growth factors and/or cytokines to modulate the growth and differentiation of stem cells.

### 3.3 Outcomes reported

The outcomes of the included in vitro studies are summarized in Table 3. The regeneration ability of in vitro cultured stem cells is evaluated by their chondrogenic, osteogenic and sometimes adipogenic and neurogenic potential (Bailey et al., 2007; Koyama et al., 2011; W.; Liu et al., 2017; Sun et al., 2014; Yao et al., 2018). Chondrogenic potential can be evaluated by collagen I (predominant in fibrocartilage), collagen II (Col‐II), collagen X (Col‐X), Aggrecan (glycosaminoglycan that predominates in cartilage), sex‐determining region Y‐box 9 (SOX 9) expression; safranin‐O staining (stains chondroitin‐4‐sulphate and chondroitin‐6‐sulphate of extracellular glycosaminoglycan); and ALP activity. Osteogenic potential can be evaluated by ALP activity, Von Kossa stain (for calcium deposits), osteocalcin (Bone Gamma‐Carboxyglutamate Protein, BGLAP), runt‐related transcription factor 2 (Runx‐2) expression. Adipogenic potential can be evaluated by oil red O stain, Sudan black B stain and adipocyte‐specific peroxisome proliferator‐activated receptor *g* transcript variant 2 (PPARg2), lipoprotein lipase (LPL) expression. Neurogenic potential can be evaluated by neuro‐type morphology (bipolar and stellate morphology); and Nestin, NeuN, glia fibrillary acidic protein (GFAP) expression (Bailey et al., 2007; Koyama et al., 2011; Liu et al., 2017; Sun et al., 2014; Yao et al., 2018).

**TABLE 3:** Outcomes reported on included in vitro studies

Study	Outcomes reported		
	Morphological/mechanical outcomes	Gene expression	Protein synthesize
Bailey et al. (2007)	Significantly more HUCM cells per construct than condylar cartilage cells	None	More intense GAG, chondroitin‐4 sulphate, chondroitin‐6 sulphate staining in HUCM group
			Significant increase of type I collagen and minute amounts of type II collagen in HUCM construct cultured in chondrogenic medium; weak presence of type I and type II collagen in TMJ construct
Bousnaki et al. (2018)	DPSC/scaffold significantly increase storage modulus and elastic response	DPSC/scaffold show significant increase of Col‐I, Col‐X, Sox9, Comp, aggrecan compared to hNPCs	DPSC/scaffold construct cultured in chondrogenic medium showed increased ECM deposition, GAGs production and aggrecan staining
Brady et al. (2011)	None	Osteogenic markers (ALP, BSP, RUNX2) were detected in the bone‐like construct	von Kossa staining showed mineralization of matrix by MSCs preconditioned in osteogenic media
		Chondrogenic markers (aggrecan, SOX9) were detected in cartilage‐like construct	Alcian blue staining showed GAG deposition by MSCs preconditioned in chondrogenic media
Koyama et al. (2011)	None	Chondrogenic potential: Significant increase of Col‐II and Col‐X mRNA in chondrogenic medium	Adipogenic potential: Positive oil red O staining in adipogenic medium after 28 days of culture
		Osteogenic potential: Significant increase of BGLAP mRNA in osteogenic medium	Neurogenic potential: Synovial fluid derived cells acquired a bipolar and stellate morphology and expression of Nestin and NeuN
Legemate et al. (2016)	No change of tensile modulus with growth factors. Significantly increased compressive modulus by higher dose of growth factors in both AP bands and IZ.Both instantaneous and relaxation moduli were significantly lower with high dose growth factorsCoefficient of viscosity was significantly higher with growth factors	Both high and low dose of growth factors significantly increase Col‐I mRNA	Growth factor formed collagen I rich fibrocartilagious matrix throughout scaffolds and collagen type I/aggrecan fibrocartilaginous matric in IZ
			Total collagen in AP bands increase with increased dose of growth factors compared to no growth factors
			Total GAG in IZ increase with increased dose of growth factors compared to no growth factors
			Both high and low dose of growth factors increase Col‐II/Aggrecan fibrocartilagious tissue in IZ but not in AP bands
Liu et al. (2017)	Cytokines expression of IL‐8, IL‐1b, IL‐6, IL‐10, TNF‐a, and IL‐12p levels quantified	In osteogenic medium, expression of RUNX2 and OCN were significantly increased	Alizarin red stain of calcium deposition found in osteogenic medium but not in control medium
		In adipogenic medium, expression of PPARG2 and LPL were significantly increased	Oil red O‐positive in lipid‐laden cells in adipogenic medium
			Cartilage pallets in chondrogenic medium show Safranin‐O stain positive and extensive immunoactivity for collagen II but not in control medium
Sun et al. (2014)	Proliferation rate: Synovium fragment‐derived cell proliferation was observed after a few days in culture. Cell growth pattern was similar to synovial fluid derived MSCs	Osteogenic differentiation: Runx2 upregulated in osteogenic induction medium.	In osteogenic induction medium, calcium deposits confirmed by von Kossa and Alizarin red staining.
		Adipogenic differentiation: LPL unregulated in adipogenic induction medium	In adipogenic induction medium, oil red O‐positive and Sudan black‐B positive, lipid‐laden cells were found
			In chondrogenic induction medium, cartilage nodules confirmed by collagen type II
			In neurogenic induction medium, cells showed Nestin and GFAC positivity
Yao et al. (2018)	Cell proliferation: SFCs exhibited similar growth pattern as SSSCs	Osteogenic, adipogenic and chondrogenic differentiation: Expression of RUNX‐2, OCN, ALP, PPARG2, LPL, SOX9, Col‐II were significantly upregulated in respective induction medium	Calcium deposits is confirmed by Alizarin Red S staining in osteogenic medium
	The clone‐forming rate of SFCs was slightly lower than SSSCs		Oil red O‐positive, lipid‐laden fat cells were identified in adipogenic medium
			Production of GAG was significantly upregulated in chondrogenic medium
Zhang et al. (2014)	PD‐MSCs disappear after co‐culture	Conditioned medium of PD‐MSCs increased expression of Col‐I, Col‐II and aggrecan significantly; increase of Sox‐9 was not significant	Mixture group contained more GAG than TMJ fibrochondrocytes alone

Abbreviations: ALP, alkaline phosphatase; AP, anterior‐posterior bands; BGLAP, bone gamma‐carboxyglutamate; BSP, bone sialoprotein; Col‐I, collagen type I; Col‐II, collagen type II; Col‐X, collagen type X; Comp, cartilage oligomeric matrix protein; DPSC, dental pulp stem cells; ECM, extracellular matrix; GAG, glycosaminoglycans; GFAC: glial fibrillary acidic protein; HUCM, human umbilical cord matrix; IZ, intermediate zone; LPL, lipoprotein lipase; NeuN, neuronal nuclear protein; OCN, osteocalcin; PD‐MSCs, periodontal ligament‐derived mesenchymal stem cells; PPARG2, peroxisome proliferator‐activated receptor‐gamma; RUNX2, RUNX family transcription factor 2; SFCs, synovial fragment cells; Sox‐9, SRY‐box transcription factor 9; SSSCs, surgery‐obtained synovium specimen cells; TMD, temporomandibular disorders.

### 3.4 Summary of results reported on in vitro studies

Results reported on included in vitro studies for ALP activity, Glycosaminoglycan (GAG) content, collagen I (Col‐I), relative Col‐I, Col‐II, relative Col‐X, SOX9, RUNX2, LPL, and Aggrecan mRNA expression in the stem cell‐based intervention groups and control groups are reported in Table 4. As compared to controls, ALP activity was significantly increased in stem cells cultured in chondrogenic or osteogenic medium (Koyama et al., 2011; Sun et al., 2014). Significant increases in GAG content were observed by two studies (Legemate et al., 2016; Zhang et al., 2014) but not Bailey et al. (2007), who found no significant difference of GAG content of HUCM cultured in chondrogenic medium and control medium. Col‐I mRNA levels were increased in dental pulp stem cells in a chondrogenic medium as compared to an expansion medium (Bousnaki et al., 2018). Human MSC cultured with growth factors showed increased Col‐I relative mRNA in the outer band of scaffolds, while no difference was noted in inner bands with or without growth factors (Legemate et al., 2016). Periodontal ligament‐derived MSC‐conditioned medium increased Col‐I relative mRNA level of chondrocytes (Zhang et al., 2014). Relative Col‐II mRNA expression and Col‐X mRNA expression were increased in both studies that tested it (Bousnaki et al., 2018; Koyama et al., 2011; Zhang et al., 2014). Relative SOX9 mRNA expression was increased in the studies by Bousnaki et al. (2018) and Zhang et al. (2014). Relative RUNX2 mRNA expression was increased in studies by Liu et al. (2014) and Sun et al^.^ (2014). Brady et al. (2011) also examined the expression of SOX9 and RUNX, without the control group. Finally, relative LPL expression was increased in both studies that tested it (W. Liu et al., 2017; Sun et al., 2014).

**TABLE 4:** Results reported on included in vitro studies for ALP activity, GAG content, Col‐I, relative Col‐I/Coll‐II/Col‐X/SOX9/RUNX2/LPL/Aggrecan mRNA expression in the stem cells‐based intervention groups and control groups

Study	Stem cells group	Rel exp	*n*	Control group	Rel exp	*n*	Estimated risk ratio	*p*‐value in study
**ALP activity**								
Koyama et al. (2011)	Induction (Chondrogenic media)	1.45	3	Control	0.35	3	4.14	*p* < 0.05
Koyama et al. (2011)	Induction (Osteogenic media)	0.98	3	Control	0.27	3	3.63	*p* < 0.05
Sun et al. (2014)	SFMSCs	41.83	3	Control	6.11	3	7.10	*p* < 0.01
Sun et al. (2014)	SFCs	46.68	3	Control	7.05	3	6.90	*p* < 0.01
**GAG content**								
Bailey et al. (2007)	HUCM chondrogenix	3.03	4	HUCM control	1.75	4	1.73	NS
Legemate et al. (2016)	IZ with GF	3.64	5	IZ without GF	0.90	5	4.04	*p* < 0.05
Legemate et al. (2016)	AP with GF	2.08	5	AP without GF	0.88	5	2.36	*p* < 0.05
Zhang et al. (2014)	Conditioned medium	8.34	6	Expansion medium	6.00	6	1.39	0.0164
**Col‐I mRNA**								
Bousnaki et al. (2018)	STEM PRO D7	3085	3	CCMD7	1.00	3	3085	0.0002
Bousnaki et al. (2018)	STEM PRO D14	5417	3	CCMD14	42.10	3	128.67	*p* < 0.0001
Bousnaki et al. (2018)	STEM PRO D21	3349	3	CCMD21	99.70	3	33.59	*p* < 0.0001
**Relative Col‐I mRNA expression**								
Legemate et al. (2016) **(50 mg)**	Growth factors ‐ I	1.76	5	No growth factor‐I	1.02	5	1.73	NS
Legemate et al. (2016) **(50 mg)**	Growth factors ‐O	8.01	5	No growth factor‐O	2.10	5	3.81	*p* < 0.05
Legemate et al. (2016) **(100 mg)**	Growth factors‐I	1.76	5	No growth factor‐I	2.85	5	0.62	NS
Legemate et al. (2016) **(100 mg)**	Growth factors‐O	8.48	5	No growth factor‐O	2.44	5	3.48	*p* < 0.05
Zhang et al. (2014)	Conditioned medium	4.20	4	Expansion medium	1.01	4	4.16	0.0027
**Relative Col‐II mRNA expression**								
Koyama et al. (2011)	Induction	2.22	3	Control	0.16	3	13.88	*p* < 0.05
Zhang et al. (2014)	Conditioned medium	2.35	4	Expansion medium	0.99	4	2.37	0.0260
**Relative Col‐X mRNA expression**								
Bousnaki et al. (2018)	Dental pulp stem cells	1.70	3	Human nucleus pulposus cells	0	3	0	0.0016
Koyama et al. (2011)	Induction	1.88	3	Control	0.31	3	6.06	*p* < 0.05
**Relative SOX9 mRNA expression**								
Bousnaki et al. (2018)	Dental pulp stem cells	10.00	3	Human nucleus pulposus cells	45.40	3	0.22	0.0180
Zhang et al. (2014)	Conditioned medium	1.41	4	Expansion medium	1.00	4	1.41	0.36
**Relative RUNX2 mRNA expression**								
Liu et al. (2017)	Induction	11.95	19	Control	0.98	19	12.19	*p* < 0.05
Sun et al. (2014)	SFMSCs	1.87	3	Control	1.01	3	1.85	*p* < 0.05
Sun et al. (2014)	SFCs	2.45	3	Control	0.62	3	3.95	*p* < 0.01
**Relative LPL mRNA expression**								
Liu et al. (2017)	Induction	427.14	19	Control	0.01	19	42,714.00	*p* < 0.05
Sun et al. (2014)	SFMSCs	117.00	3	Control	0.33	3	354.55	*p* < 0.01
Sun et al. (2014)	SFCs	108.00	3	Control	9.33	3	11.58	*p* < 0.01
**Relative aggrecan mRNA expression**								
Zhang et al. (2014)	Conditioned medium	1.69	4	Expansion medium	1.00	4	1.69	0.0059

*Abbreviations:* ALP, alkaline phosphatase; AP, anterior‐posterior band; Col‐I, collagen I; Col‐II, collagen II; Col‐X, collagen X; GAG, glycosaminoglycans; GF, growth factor; Growth factors‐I, Growth‐factors intermediate zone; Growth factors‐O, growth factors outer band; HUCM, human umbilical cord matrix; IZ, intermediate zone; LPL, lipoprotein lipase; mRNA, messenger RNA; NS: none significant; RUNX2, RUNX family transcription factor 2; SFCs, synovial fragment cells; SFMSCs, synovial fragment mesenchymal stem cells; STEM Pro D7, Thermo Fisher Scientific chondrogenic medium day 7; STEM Pro D14, Thermo Fisher Scientific chondrogenic medium day 14; STEM Pro D21, Thermo Fisher Scientific chondrogenic medium day 21; SOX9, SRY‐box transcription factor 9.

### 3.5 Meta‐analyses

Review authors could not conduct meta‐analyses as the authors did not report Odds ratios or Risk ratios with 95% confidence intervals for the outcomes of interest in the stem cells groups compared to control groups on ALP activity, GAG content, Col‐I, relative Col‐I, Col‐II, Col‐X, SOX9, RUNX2, LPL, and Aggrecan mRNA expression.

## 4 DISCUSSION

Large joints' regeneration is challenging due to the limitations of tissue engineering techniques and the complex anatomy of large joints. However, TMJ might be the first to benefit from current advances of tissue regeneration due to its small size (Brady et al., 2011). Although TMJ regeneration is still at its early stage, and a few pioneer studies have used stem cells to treat TMD patients (Carboni et al., 2019; De Riu et al., 2019; Howlader et al., 2017), most TMJ regeneration studies were conducted in vitro.

### 4.1 Human umbilical cord‐derived MSC

Bailey et al. (2007) compared polyglycolic acid (PGA) scaffolds seeded with human umbilical cord‐derived mesenchymal‐like stem cells (HUCM) and PGA scaffolds seeded with TMJ condylar chondrocytes. Authors found that after 4 weeks of culture, HUCM scaffolds contain more collagen I and GAG protein content than chondrocytes scaffolds (Bailey et al., 2007).

### 4.2 Synovial fluid‐derived cells

Koyama et al. (2011) first characterized synovial fluid‐derived cells as multipotent stem cells. These cells were fibroblastic spindle‐shaped and expressed Stro‐1 and CD146, usually presented in bone marrow mesenchymal cells. Authors found that these cells showed multipotency under different conditions that mimic bone marrow‐derived MSCs. In a chondrogenic induction medium, cartilage nodules can be formed with a positive toluidine blue stain. Also, RT‐PCR showed increased Coll‐II and Col‐X. In an osteogenic induction medium, calcium deposits can be formed with a positive von Kossa stain, RT‐PCR revealed increased BGLAP. In adipogenic induction medium, cells developed into oil red‐O‐positive lipid‐laden fat cells with increased expression of PPARg2 and LPL as detected by RT‐PCR. In neurogenic induction medium, cells differentiated to bipolar and stellate forms with increased expression of Nestin and NeuN (Koyama et al., 2011).

Sun et al. (2014) and Yao et al. (2018) demonstrated that synovium fragment‐derived cells (SFCs) and surgery‐obtained synovium specimen cells (SSSs) had similar morphological features and multi‐lineage differentiation potential as synovial fluid‐derived MSCs (SFMSCs). However, in these studies, SFCs, SSSs, and SMSCs were Stro‐1 and CD146 negative, which could be caused by the instability of STRO‐1 and CD146 expression in MSCs and the differences between donor and culture conditions.

Currently, there is no consensus regarding markers for SFMSCs or tissue of origin of these cells (Sun et al., 2014; Yao et al., 2018). According to few studies, intra‐articular bleeding and early stages of osteoarthritis can increase synovial fluid MSC (Koyama et al., 2011; Sun et al., 2014). Jones et al. (2004) proposed that disrupted joint structures may be the source of these cells. Sun et al. (2014) suggested that disrupted articular cartilage or bones may not be the origin of SFMSCs in TMJ because they obtained SFMSCs from patients with TMD without tissue damage. In addition, SFCs with almost identical characteristics as SFMSCs were primarily from intima of TMJ, which emphasize that SFMSCs could be from TMJ intima as well. SFCs could be a source for MSC‐based TMJ regeneration (Sun et al., 2014; Yao et al., 2018). Liu et al. (2017) further evaluated how inflammation affects the multi‐lineage differentiation potential of SFCs. TMDs are inflammatory conditions in which various pro‐inflammatory cytokines can be found. Liu et al. (2017) characterized the cytokines in synovial fluid of 19 patients with TMD and found that IL‐8, IL‐1b, IL‐6, IL‐10, TNF‐a, and IL‐12r levels were elevated as patients' dysfunctional index increased. They further demonstrated that IL‐1b significantly increased IL‐6 and IL‐8 levels through IL‐1b‐dependent nuclear factor‐kB (NF‐kB) pathway activation. Also, authors observed that IL‐1b impeded chondrogenic differentiation of SFMSCs. Pre‐exposure to IL‐1b or IL‐6 downregulated SOX9 expression and reduced glycosaminoglycan content of SFMSCs. Liu et al. (2017) results suggested that MSCs have no beneficial effect in some arthritis models, but, rather, aggravated arthritis symptoms (Djouad et al., 2005).

### 4.3 Dental pulp stem cells

In contrast to the above papers that studied multi‐lineage differentiation potential of various stem cells, Bousnaki et al. (2018) and Legemate et al. (2016) studied stem cells seeded in engineered scaffolds and the possibility of TMJ disc replacement with tissue engineering. Bousnaki et al. (2018) evaluated TMJ disc regeneration by seeding chitosan/alginate scaffolds (Ch/Alg I and Ch/Alg II) with dental pulp stem cells (DPSCs) and intubated for 8 weeks. DPSCs share embryonic origin with TMJ discs. Scaffolds seeded with human nucleus pulposus cells (hNPCs) were used as control. Two different scaffolds were compared: Ch/Alg I scaffold was crosslinked in a solution of 4% w/v CaCl_2_ in ultrapure water; Ch/Alg II scaffold was crosslinked in a solution of 4% w/v CaCl_2_ and 0.1% v/v glutaraldehyde. They assessed cell attachment, viability and proliferation in two scaffolds, along with, the chondrogenic potential of DPSCs and thermomechanical properties under compression of regenerated DPSC/scaffold construct and observed that the extracellular fibrocartilaginous matrix production significantly increased in stiffer and more crosslinked Ch/Alg‐II scaffolds and chondrogenic medium further increased fibro/chondrogenic differentiation. However, RT‐PCR of chondrogenic markers such as ACAN, COMP, COLI, COLX, SOX9, and ACAN were not increased in Ch/Alg‐I scaffold, even though HE and Alcian blue showed ECM deposition in both scaffolds. Also, the thermomechanical characters of both scaffolds were similar to native TMJ disc. The authors concluded that hybrid Ch/Alg II scaffolds can support stem cell growth and provided a favored fibro/chondrogenic differentiation of DPSCs (Bousnaki et al., 2018).

### 4.4 Bone marrow‐derived stem cells

Legemate et al. (2016) developed a 3‐dimensional (3D)‐printed scaffold to replicate anisotropic collagen orientation of fibrocartilaginous matric distribution of human TMJ disc. The authors aimed to reproduce the region‐dependent anisotropic tensile properties of the human TMJ disc with this specific design to restore function better. The scaffold was cultured with human MSCs, connective tissue growth factor and transforming growth factor‐beta 3 containing microspheres (CTGF/TGFβ3‐μS). Due to the different orientation of collagen fibers, the engineered scaffold was analyzed in both anterior/posterior (AP) and intermediate zones (IZ) (Legemate et al., 2016).

Legemate et al. (2016) observed that culturing with MSCs for 6 weeks with CTGF/TGFβ3‐μS resulted in collagen‐rich fibrous structure distribution in the AP bands and collagen type I/aggrecan rich fibrocartilaginous matrix deposition in the intermediate zone. High doses of CTGF/TGFβ3‐μS resulted in denser collagenous tissue in AP bands and denser cartilaginous matrix in IZ as compared to low doses of CTGF/TGFβ3‐μS. Although there was no significant difference in tensile modulus in scaffold with CTGF/TGFβ3‐μS microsphere or empty microsphere, a higher dose of CTGF/TGFβ3‐μS resulted in significantly higher compressive modulus and coefficient of viscosity in AP bands and lower compressive modulus in IZ, along with, lower instantaneous and relaxation moduli in both AP and IZ bands. These characteristics are almost similar to the native TMJ disc (Legemate et al., 2016). Bousnaki et al. (2018) and Legemate et al. (2016) demonstrated the construction of scaffolds that mimic native TMJ disc for the growth/differentiation of stem cells. However, in vivo studies are needed to assess their performance in humans.

In contrast to the above TMJ disc regeneration studies, Brady et al. (2011) tried to engineer a biphasic osteochondral construct to create a structure similar to native TMJ condylar tissue for in vivo implantation and established a biphasic matrix with hyper‐hydrated collagen gel. Iliac crest‐derived bone marrow MSCs preconditioned in osteogenic and chondrogenic media were seeded in the biphasic‐compressed gel. The construct was cultured for 7 days before osteo‐ and chondro‐differentiation were analyzed (Brady et al., 2011). Authors found that a week after culture, distinct bone‐like and cartilage‐like zones were identified in the compressed biphasic matrix, as demonstrated by von Kossa staining, Alcian blue staining, and expression of ALP, BSP, RUNX2, aggrecan, and SOX‐9. No significant mixing of the two preconditioned cell types was found (Brady et al., 2011).

### 4.5 Periodontal ligament derived stem cells

Finally, Zhang et al. (2014) studied the supportive effects of periodontal ligament MSCs (PD‐MSCs) on fibrochondrocytes from TMJ disc. A prior study by Wu et al. (2011) had shown that coimplanted articular chondrocytes and MSCs increased chondrocytes proliferation and matrix formation. PD‐MSCs are easily accessible to oral surgeons, while fibrochondrocytes from TMJ disc have a limited source of supply. Zhang et al. (2014) observed significant increases in proliferation of chondrocytes, GAG deposition, and expression of Col‐I, Col‐II, and Aggrecan during co‐culture of PD‐MSCs with fibrochondrocytes and also the absence of PD‐MSC at 3 weeks. PD‐MSCs conditioned medium revealed effects similar to that of co‐culture of PD‐MSC with fibrochondrocytes. Thus, they concluded that the interaction between PD‐MSCs and fibrochondrocytes were mediated through soluble factors secreted by PD‐MSCs, such as basic bFGF, vascular endothelial growth factor A (VEGF‐A), interleukin‐6 (IL‐6), and interleukin 8 (IL‐8) (Zhang et al., 2014).

## 5 CONCLUSIONS

In summary, in vitro studies utilized several different types of stem cells under different conditions. Increased osteogenesis and/or chondrogenesis were noted with stem cell interventions compared to control groups on ALP activity, Col‐I, Col‐II, Col‐X, RUNX2, LPL, and Aggrecan mRNA expression. This review emphasizes the potential of stem cell therapies in the regeneration of TMJ‐related structures. However, due to the heterogeneity of stem cells used, experimental conditions, outcome assessments and the limited number of studies available, we could not conclude that one type of human stem cells or a culturing condition is better than others. The field of TMJ regeneration using stem cell therapy is still at its infancy and further RCTs are required in order to evaluate the efficacy and safety of these therapies in humans.

## CONFLICT OF INTEREST

The authors of this review had no conflicts of interest.

## AUTHOR CONTRIBUTIONS

Dr. Shan Gong contributed the conceptualization, data acquisition, statistical analyses and the writing of the manuscript. Dr. Kamal Al‐Eryani contributed by supervising this work, reviewed and revised the manuscript, and contributed to the final version of the manuscript. Dr. Emperumal contributed to conceptualization, drafting and review. Dr. Enciso contributed the conceptualization, data acquisition, statistical analyses and the writing/revisions of the manuscript.

## Data Availability

Data sharing is not applicable to this article as no new data were created or analyzed in this study.
